# Parametric model fitting-based approach for retinal blood vessel caliber estimation in eye fundus images

**DOI:** 10.1371/journal.pone.0194702

**Published:** 2018-04-18

**Authors:** Teresa Araújo, Ana Maria Mendonça, Aurélio Campilho

**Affiliations:** 1 Faculdade de Engenharia da Universidade do Porto (FEUP), 4200-465 Porto, Portugal; 2 Instituto de Engenharia de Sistemas e Computadores - Tecnologia e Ciência (INESC-TEC), 4200 Porto, Portugal; University of Arizona, UNITED STATES

## Abstract

**Background:**

Changes in the retinal vessel caliber are associated with a variety of major diseases, namely diabetes, hypertension and atherosclerosis. The clinical assessment of these changes in fundus images is tiresome and prone to errors and thus automatic methods are desirable for objective and precise caliber measurement. However, the variability of blood vessel appearance, image quality and resolution make the development of these tools a non-trivial task.

**Metholodogy:**

A method for the estimation of vessel caliber in eye fundus images via vessel cross-sectional intensity profile model fitting is herein proposed. First, the vessel centerlines are determined and individual segments are extracted and smoothed by spline approximation. Then, the corresponding cross-sectional intensity profiles are determined, post-processed and ultimately fitted by newly proposed parametric models. These models are based on Difference-of-Gaussians (DoG) curves modified through a multiplying line with varying inclination. With this, the proposed models can describe profile asymmetry, allowing a good adjustment to the most difficult profiles, namely those showing central light reflex. Finally, the parameters of the best-fit model are used to determine the vessel width using ensembles of bagged regression trees with random feature selection.

**Results and conclusions:**

The performance of our approach is evaluated on the REVIEW public dataset by comparing the vessel cross-sectional profile fitting of the proposed modified DoG models with 7 and 8 parameters against a Hermite model with 6 parameters. Results on different goodness of fitness metrics indicate that our models are constantly better at fitting the vessel profiles. Furthermore, our width measurement algorithm achieves a precision close to the observers, outperforming state-of-the art methods, and retrieving the highest precision when evaluated using cross-validation. This high performance supports the robustness of the algorithm and validates its use in retinal vessel width measurement and possible integration in a system for retinal vasculature assessment.

## 1 Introduction

The retina is a light-sensitive tissue that converts the incoming light into neural signals that are interpreted by the brain. Adequate techniques, such as color fundus photography, allow to non-invasively assess the retina and its structures. Namely, the retinal blood vessels are the only portion of the circulatory system that is directly observable and thus the study of their morphological changes has been associated with a variety of conditions and their risk [1, 2]. For instance, changes in retinal vessel caliber are an important sign of diabetes mellitus, hypertension, arteriosclerosis and cardiovascular diseases [3, 4], as well as pre-diabetes and pre-hypertension [2, 5]. Consequently, vessel width alterations could be used for prevention and diagnosis. The early diagnosis of these mentioned diseases is crucial to prevent and reduce health damages. Due to the effort and time that would be required to manually measure the vessels calibers at a large portion of the vasculature, this option is currently unfeasible in clinical practice. With that in mind, automated segmentation and measurement of the vessels is desirable, since it would enable the systematic evaluation of the vessel width changes that could be useful for diagnosis, and contribute to the efficiency, reliability and reproducibility of the measurements. Automatic methods are particularly useful in wide screening programs for vascular conditions, since the human analysis of a large number of images together with the complexity of the retinal vascular network is a heavy task. Consequently, there is the need for developing computer-aided diagnosis (CAD) systems to help in the quantification of retinal structures and biomarkers assessment. Several authors stress that the vessel width measurement stage is sufficiently critical to be individually and carefully studied [6, 7]. The development of automated methods for width measurement is a demanding process, considering: 1) the variability of the appearance of blood vessels; 2) the variability of image quality and resolution and 3) the lack of standardized data and criteria for comparing algorithms, preventing significant comparisons in large scale [8].

### 1.1 State-of-the-art methods

State-of-the art methods for retinal vessel width measurement can be grouped in two major schemes, as proposed in [8]: methods based on vessel contour detection or on parametric model fitting. The first type of methods generally measure the diameter from the vessel contours and thus heavily rely on the contrast between vessel and the background. Common factors that can hinder the segmentation are the presence of other anatomical structures, low contrast and image artifacts. For that, active contours [9], graphs [10], wavelets [11] and tracking [12, 13] can be used. Whilst some authors measure the vessel caliber directly from the segmented vessels, others use the segmentation solely as a starting point, using it for vessel centerline determination or for having an initial estimation of the widths [11]. Bankhead *et al*. [11] proposed a method for vessel detection and diameter measurement using wavelet-based segmentation and edge refinement. This involves centerline computation and refinement from the segmented vessels, cross-section vessel profile generation and vessel edge identification based on gradient information.

Methods that fit parametric models to the vessel cross-section intensity profiles rely on the found parameters to determine the vessel widths. The general approach usually starts by vessel segmentation, centerline detection and removal of junctions to obtain individual vessel segments. Then, vessel cross-section intensity profiles are computed, and a parametric intensity model is fitted to the profile. The determination of the vessel widths is based on the parameters of the best-fit profile. Alternatively, some approaches do not perform vessel detection and instead measure the width at specific points [8]. Commonly, these methods rely on the expected Gaussian-like shape of the vessel cross-section intensity profile [14, 15]. However, central light reflex (CLR) alters the shape of the Gaussian by creating a high intensity peak on the center region of the profile [16, 17] (S1 Appendix). CLR can be accounted for by using, for instance, piece-wise Gaussian models [18] and Difference-of-Gaussian-based models [6, 16]. Further, vessels can present an asymmetrical shape [19]. Unlike most studies, Lupascu *et al*. [8] proposed an Hermite model, adapted from the approach introduced in [20], that considers both CLR and vessel asymmetry, but the asymmetry is restricted to the vessel center region and does not significantly affect the vessel limits. Parametric model fitting approaches can be 1D or 2D if one or multiple cross section intensity profiles are considered, respectively. From these, 2D approaches are more robust against noise. The determination of the width from the best-fit model’s parameters is usually done using fixed scaling factors [6, 14, 21, 22]. For instance, Zhou *et al*. [14] fitted a 1D-Gaussian function to the vessel profile and estimated the vessel diameter by multiplying the spread of the best fit model by a constant equal to 3.92. More sophisticated approaches have been recently proposed, applying supervised learning to find the relationship between the model’s parameters and the vessel diameter [8]. Most of the state-of-the-art methods present limitations, such as poor performance in low resolution images or thin vessels, as well as susceptibility to artifacts and pathologies.

### 1.2 Contributions

The herein proposed vessel caliber measurement method contributes to the state-of-the-art as follows:

1. Novel parametric models for vessel intensity profile fitting. These are modified Difference-of-Gaussian models where a multiplying line with varying slope modulates the asymmetry of the vessel edges, thus allowing the adjustment of a large variety of vessel profiles. The performance of the models is extensively evaluated and proves to outperform other known models for fitting of vessel cross-section intensity profiles;
2. A top performing model fitting-based approach for retinal vessel width estimation. The method combines model fitting with multiple preprocessing steps, estimating the vessel diameter using ensembles of bagged regression trees with random feature selection. The combination of different approaches makes the algorithm robust and reliable for width estimation in images with pathologies and artifacts, with performance independent of the true vessel widths. The results are close to the medical gold standard and often outperform the state-of-the-art methods.

This study is an extension of our previous work [23], in which we first proposed one of the herein presented parametric models and the approach for vessel width estimation. Here we present another version of this model and evaluate the models’ goodness of fit to the vessel cross-section profiles. Besides being more focused on the fitting component of the method than the previous publication, the overall performance of the width estimation method is also explored in detail. Additionally, we have developed a graphical user interface for the proposed method, which was implemented in MATLAB (S1 Video), and is available at https://rdm.inesctec.pt/dataset/nis-2018-002.

This document is divided as follows: in section 2 the novel method for retinal vessel width estimation is described. Section 3 presents and discusses the experimental results. Finally, in section 4 the main conclusions of this work are presented, as well as suggestions for future work.

## 2 Method for retinal blood vessel width estimation

**Fig 1:**
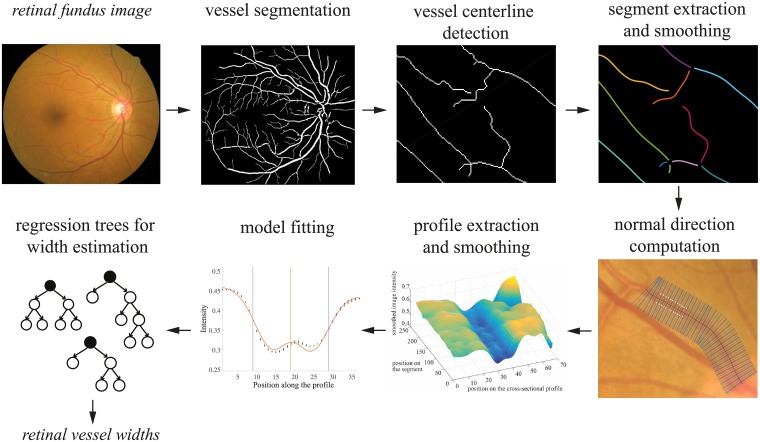
Method overview. Retinal blood vessels are segmented and their centerlines are detected, followed by junction removal to extract segments which are then smoothed. Cross-section intensity profiles are extracted perpendicularly to the centerlines and model fitting is performed on smoothed profiles. Based on the best-fit model parameters, vessel width is estimated through regression.

The proposed method for retinal blood vessel width estimation follows a model fitting-based approach, including several profile processing steps prior to model fitting. Then, ensembles of bagged regression trees are used for estimating the vessel diameters from the best-fit model parameters. An overview of the different phases involved in the algorithm is shown in Fig 1. First, vessels are segmented from the eye fundus image and the respective centerlines are obtained through thinning. Each vessel segment is then smoothed through spline approximation and, for each segment pixel, the intensity profile normal to the segment at that point is extracted. These profiles, spatially smoothed to reduce noise, are used for the parametric model fitting. We propose a new parametric model for the cross-section intensity profiles, where the parameters are estimated through least square minimization. Then, the best-fit model parameters are the input of a random forest regression system, that allows the estimation of the vessel width, exploring the embedded relationship between the width and the model parameters.

### 2.1 Vessel segment extraction

**Fig 2:**
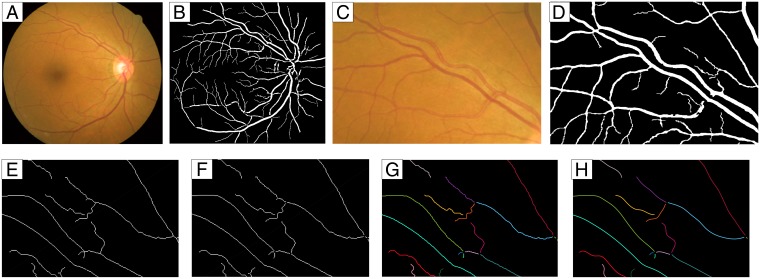
Blood vessel segmentation, centerline detection, and segment extraction and smoothing. Top row: example of vessel segmentation; second row: example of removal of junctions from a thinned vessel image and vessel segment smoothing through spline fitting. **A**: image from REVIEW dataset (CLRIS001); **B**: segmented image [24]; **C**: region from [A]; **D**: region from [B]; **E**: thinned vessels for a region of [D]; **F**: vessel segments, after junction removal; **G**: vessel segments of [F], labeled with different colors; **H**: vessel segments of [G] after spline approximation. Colors are used for better distinguishing between vessel segments.

Vessel segment extraction starts from the segmentation of the retinal vasculature using a morphology-based state-of-the-art approach [24] (Fig 2). Then, vessel centerlines are detected using a thinning technique [25, 26] followed by the removal of bifurcation and crossover points by analyzing the number of neighbors of each pixel. This divides the whole centerline network into individual segments where the diameters will be measured. These segments correspond to a pixel-thin group of connected pixels limited by two end points, i.e., pixels with a single neighbor. Fig 2E and 2F show an example of thinning and junction removal steps.

The obtained segments are then refined by removing short spurs that may have resulted from the thinning process. All terminal segments, i.e., that contain end points, shorter than 10 pixels are removed [11]. These structures usually result from irregularities on the vessel segmentation and thus are not of interest. Longer structures are kept because they may correspond to short vessel segments.

### 2.2 Vessel cross-section intensity profile determination

Once vessel segments are found, intensity profiles are determined perpendicularly to the vessel centerlines. For each vessel segment, at each center point, the intensity values along the normal to the centerline at that point are obtained. In order to do this, we use a process similar to the one presented in [11].

#### 2.2.1 Vessel segment smoothing

**Fig 3:**
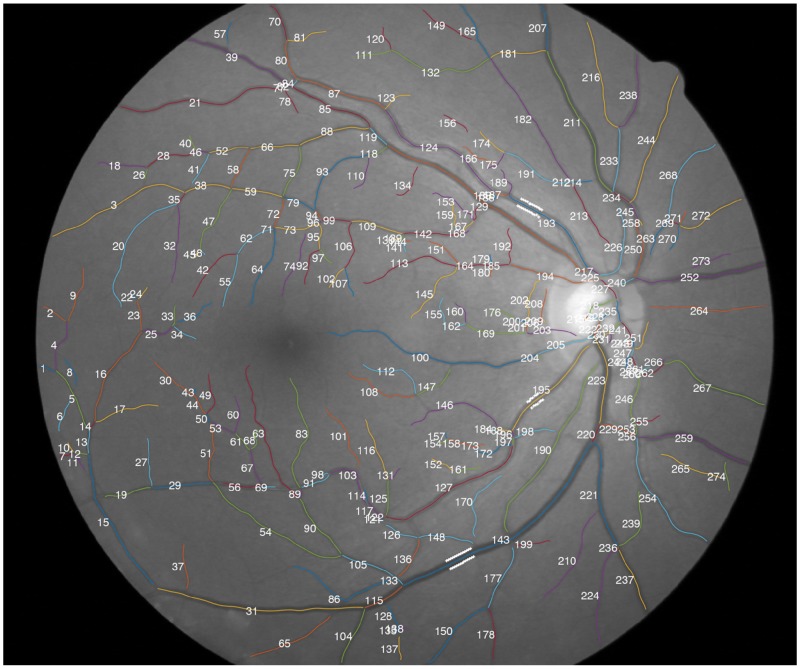
Blood vessel segments obtained for CLRIS001 image (REVIEW dataset). Segments are numbered, colored and overlapped with the green channel of the RGB image (note that different segments may be represented in the same color). White marks along some blood vessel edges represent ground truth points.

The extraction of the intensity profiles requires knowledge of the blood vessel orientation. A simple approach would be to compute the derivatives at the pixel-discrete vessel segments. However, this process may retrieve inaccurate results because very abrupt changes can occur between one pixel and its neighbor. Spline fitting is applied to each segment to smooth the vessel and thus avoid this problem [11, 27] [28–30]. Least-squares cubic spline approximation is performed, being Lee’s centripetal scheme used for parametrization [31]. The number of polynomial pieces of the spline are determined by dividing the length of the segment by 20 pixels, since this pixel spacing was found to retrieve an acceptable smoothing for the tested images. Fig 2 shows the effect of the spline fitting on the vessel centerlines, and Fig 3 shows a full image with smoothed segments.

Once the splines are fitted to the vessel segments, the new smoothed segment points are retrieved and the first derivatives of the splines at these points are computed. From the direction of the vessel at a given point, the normal at that point can be determined.

#### 2.2.2 Profile extraction

**Fig 4:**
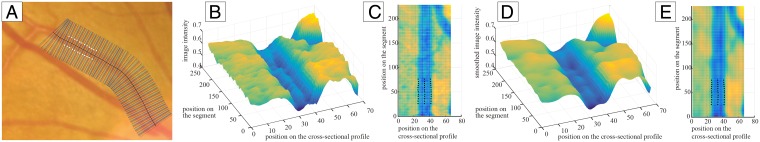
Vessel intensity profile determination and smoothing, for one segment from Fig 3. **A**: profile directions; **B**: segment intensity profiles stacked in parallel; **C**: top view of [B]; **D**: smoothed intensity profiles; **E**: top view of [D]. Colors in the plots are representative of the intensity values: warmer colors represent higher intensity whilst cooler colors represent lower intensity. The white marks in [A] and the black marks in [C] and [E] represent the ground truth annotations.

The intensity profiles along the normals to the segments are determined (Fig 4A) on the green channel of the RGB image due to the high contrast between vessel structures and the background. The intensities along the normal to the vessel are obtained with 1 pixel spacing and by applying bilinear interpolation of the intensities at non-integer locations.

The length of the profiles for a given image is determined based on the binary vessel segmentation mask, thus guaranteeing that this length is larger than the largest vessel of the image. Fig 4B shows a surface constituted by the 1D profiles extracted from the segment of Fig 4A, stacked together in parallel to each other, aligned by their center points. In Fig 4C the top view of the surface is shown, along with the ground truth marked by the observers. This image is a straightened vessel image, where all the profiles of the segment lay horizontally.

#### 2.2.3 Determination of profile lengths

Since the obtained intensity profiles may include more than one vessel, if they are close to each other, the region containing only the vessel of interest must be detected prior to model fitting. For that, a method based on peak search on the intensity profiles is applied to the mean of the vessel profiles along 11 adjacent sections (5 at each side) since the averaged profiles offer less noise than the individual one [8]. The mean profile is then smoothed using Savitzky-Golay filtering [32], and the minima and maxima in the resulting profile are detected.

**Fig 5:**
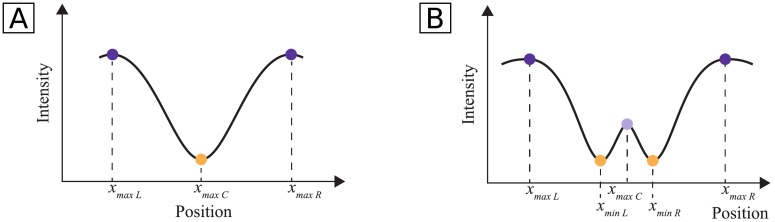
Typical shape of a blood vessel with and without central light reflex (CLR). **A**: blood vessel without CLR; **B**: blood vessel with CLR. The extreme point positions are also shown.

The profile length determination is performed in two steps, since a simple search for a typical vessel region, i.e., two maxima adjacent to a minimum, would not account for the possible existence of central light reflex (CLR), resulting in wrong detections on vessels with this characteristic. The profile of a vessel with CLR can be characterized as a region containing a maximum with one adjacent minimum and maximum on each side (Fig 5B). A search for CLR regions in the profile is performed under a set of validity rules, explained in the following paragraphs. If no CLR vessel is found, vessel regions without CLR are searched (Fig 5A). The minimum closest to the vessel center and the adjacent maximum on each side are found and validated. In the end, the length of the profiles for a given segment is set to be equal to the median of the lengths of profiles determined for that segment.

**Fig 6:**
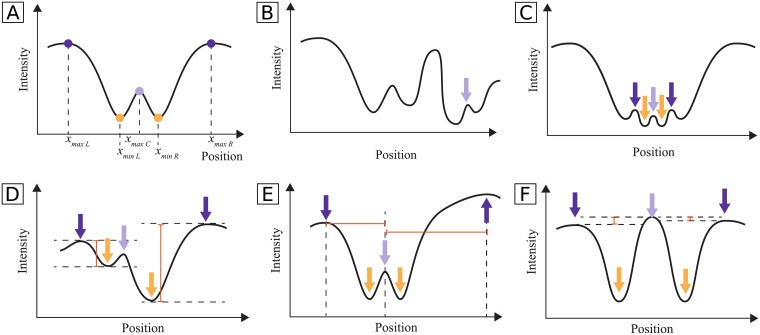
Typical shape of a vessel with CLR and cases where the conditions imposed for CLR detection are violated. **A**: typical shape of a vessel with CLR, along with its extreme point positions; **B**: the lowest maximum of the profile is not the correct CLR center; **C**: the found minima positions are too close to each other to constitute a CLR region; **D**: the two bumps of the CLR have an intensity difference larger than the acceptable; **E**: the distances between the maxima and the vessel center are too different; **F**: the elevation in the CLR center has larger intensity than the vessel limits. The arrows indicate the locations of the peaks that would define the CLR region, if one of the conditions had not been violated.

Search for CLR regions. First, the CLR center, corresponding to a maximum in the center region of the vessel, is detected. Assuming that all vessel maxima are always lower than background maxima, one can simply detect the maximum with lowest intensity, and consider its position to be the CLR center. However, other vessels may be present in the profile (see Fig 6B), possibly leading to a wrong maximum detection. To avoid this, the maximum closest to the center of the profile is detected, and chosen instead of the lowest profile maximum if it is not too far from the profile center and it is close in value to the lowest maximum.

Then, the two adjacent minima to the CLR center are detected, one to the left and one to the right of that maximum (Fig 6A). Finally, the two adjacent maxima are detected, one to the right of the right minimum and one to the left of the left maxima. These maxima positions are considered the limits of the vessel. A set of conditions is established to avoid the recognition of false CLR regions. First, the locations of the two minima should have a minimum distance. This prevents misclassifications as CLR if the peaks are too close (Fig 6C). The depths of the two bumps of the CLR should not differ too much, in order to avoid large intensity differences between the two sides of the CLR (Fig 6D). Then, the distances between the vessel center and the two maxima should not differ more than a established value (Fig 6E). Besides, the maxima should not be too far from the vessel center. If only one of the maxima is too far away from the center, what happens in the other side of the vessel is replicated, symmetrically to the vessel center. These conditions avoid a big asymmetry between the two sides of the CLR. Additionally, the elevation of the CLR center should not surpass the limits of the vessel (Fig 6F).

If no such region containing the vessel center is found, the algorithm proceeds by searching for non-CLR vessels. Otherwise, the length of the profile is computed as follows: *prof*_*length*_ = 2 × *mean*(|*x*_*maxL*_ − *x*_*maxC*_|, |*x*_*maxR*_ − *x*_*maxC*_|), where *x*_*maxL*_ and *x*_*maxR*_ correspond to the positions of the maxima that limit the vessel region and *x*_*maxC*_ to the position of the central minimum (see Fig 6A). The parameters used in these rules were obtained by experimentation, having achieved good results in the tested images.

Search for non-CLR regions. The found peaks are analysed to verify the validity of the limits. If there is no maximum to the left or to the right of the minimum, what occurs in the the other side is replicated. The two maxima must not be too close to the vessel center: if only one of the maxima is too close to the center, what happens in the opposite side is replicated; if both are too close, an iterative search for other maxima to the left and to the right of the vessel is performed until suitable maxima are found. If the above conditions are met, the length of the profile is computed as follows: *prof*_*length*_ = 2 × *mean*(|*x*_*maxL*_ − *x*_*minC*_|, |*x*_*maxR*_ − *x*_*minC*_|), where *x*_*minC*_, *x*_*maxR*_ and *x*_*maxL*_ represent the positions of the central minimum, right maximum and left maximum, respectively. Otherwise, the profile length is considered to be equal to the initial profile length.

**Fig 7:**
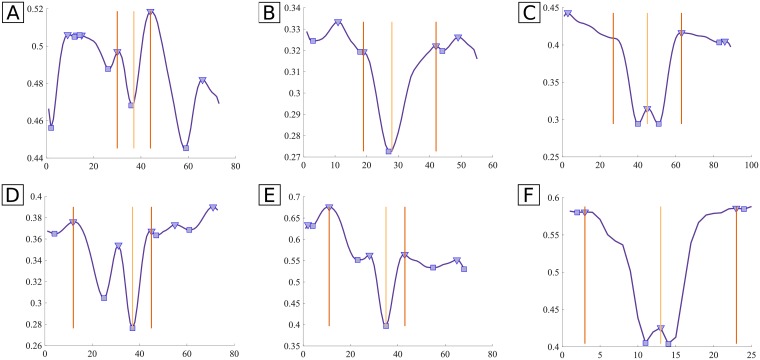
Examples of profile length determination results. Top row: successful cases; bottom row: non-successful cases, for which the conditions were not restrict enough. Curve: smoothed mean intensity profile; triangular marks: detected maxima; square marks: detected minima; orange vertical lines: detected vessel limits; yellow vertical line: center of the profile. **A** and **B**: CLR correctly rejected due to the big difference in the depth of the two depressions; **C**: left limit symmetric to the right, since it was too far away from the center; **D** and **E**: CLR wrongly detected (conditions not restricted enough); **F**: profile region overestimated (lack of peaks near the vessel limits).

In Fig 7 examples of difficult cases in which the algorithm succeeds and others in which it fails are shown. For example, in Fig 7A and 7B, the established conditions allowed to not wrongly detect a CLR. However, in Fig 7D and 7E the conditions were not restrictive enough and so a CLR was detected in a non-CLR vessel. Fig 7C shows a case where the conditions led to the replication of the right side of the vessel since the left limit was too far away from the center. Fig 7F is an example of a common problem, that is the lack of peaks near the vessel limits. This leads to an overestimation of the profile length. Note that, despite the cases where the profile length is overestimated, the length actually considered in the next steps is the median of the profiles for a given segment, and not the individual profile lengths. This means that the final length is less affected by the overestimation. Further, the overestimation, although not desirable, is preferable to the underestimation, which would lead to the loss of vessel profile information. Before model fitting, the profiles are cut, symmetrically relatively to the centerline, to the determined profile length for that segment, i.e., all profiles belonging to the a given segment have the same length.

#### 2.2.4 Profile smoothing

The obtained vessel profiles can be noisy, resulting mainly from the retinal image formation process, which can lead to poor image quality. To overcome this, a smoothing filter is applied to the segment profiles stacked in parallel to each other. A colormapped version of this straightened image is shown in Fig 4C, where one can see the all the vessel center points aligned vertically in the center column, and the profiles laying horizontally, one per row. Smoothing is performed using anisotropic Gaussian filtering, due to the different standard deviations along the two directions [11]. Since the straightened image has the profiles oriented horizontally, this filtering allows to apply a lower degree of smoothing in the direction of the cross-section profiles than in the direction of the vessel (perpendicularly to profiles), allowing to reduce noise without excessively blurring the vessel edges. The standard deviation values used are function of the estimated profile length, so that wider vessels are more smoothed than thinner ones [11]. Specifically, $\sigma_{x}=\sqrt{0.1\times prof\_length_{segment}}$ and $\sigma_{y}=\sqrt{1.5\times prof\_length_{segment}}$ are used, where *prof*_*length*_*segment*_ is the determined profile length, and *x* and *y* stand for the horizontal and vertical directions, respectively. Fig 4 shows the effect of the anisotropic Gaussian smoothing filter on a vessel segment.

### 2.3 Model fitting

The vessel intensity profiles are approximated by finding the model parameters that lead to the best fit between the model curve and the observed profile [33]. These parameters will afterwards be used for estimating the vessel widths. Different models, all with CLR-fitting capability, are herein tested. The Hermite model was selected due to its good performance on previous approaches [8]. Two new models based on Difference-of-Gaussians are proposed.

Vessel model fitting can be performed either in 1D or 2D, i.e., considering a single or multiple neighboring vessel cross-section profiles, respectively. Our approach uses 2D model fitting since it increases the robustness to noisy data by introducing some smoothing in the process, being the one used in this work. Note that these 2D models usually consist in the extrusion of a 1D model *x*, i.e., the equation of the 2D model is independent of *y* (considering the independent variables *x* and *y*). As thus, it is identical to consider a 2D model surface and fit it to the 2D cylinder of profiles or to consider the points of all the neighboring profiles (projected) in one single plane, and fit the 1D model, being that in this work we do the latter.

#### 2.3.1 Hermite model

**Fig 8:**
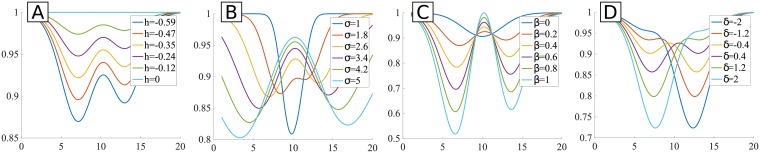
Examples of curves of the Hermite model as defined in Eq 1, but in 1D. **A**: effect of the amplitude of the main Gaussian (*h*); **B**: effect of the spread of the Gaussians (*σ*); **C**: effect of the amplitude of the two other Gaussians (*β*); **D**: effect of the CLR asymmetry (*δ*). A profile length of 20 pixels was set to the vessel. In each plot a parameter is varied at a time, with the remaining parameters fixed, in order to evaluate the influence of that parameter in the overall model shape (*t* = 1, *h* = −0.588, *β* = 0.2, *μ* = 10, *δ* = 0.2, *σ* = 2.5).

The adapted Hermite model with 6 parameters presented in [8] was first evaluated, and is defined as:

$$m\left(x,y\right)=t+h\left(1+\beta \left({\left(x-\mu -\delta \right)}^{2}-1\right)\right)\frac{1}{\sqrt{2\pi \sigma^{2}}}e^{-{\left(\frac{x-\mu }{\sqrt{2}\sigma }\right)}^{2}}$$ (1)

where *x* is the coordinate along the vessel cross-section, *y* is the coordinate along the perpendicular direction, *t* is the maximum of the function, *h* is the height of the Gaussian, *μ* the location of the center, *σ* the standard deviation of the Gaussian, *β* is an adaptive parameter controlling the depth of the concavity of the CLR and *δ* is a parameter that controls the asymmetry of the model. As referred, the 2D model can be seen as a cylinder of 1D models along the *y* axis direction and, consequently, the expression is independent of *y*. The model can also be expressed, by separating its terms, as:

$$\begin{array}{c} \begin{array}{c} m\left(x,y\right)=t+h\;\frac{1}{\sqrt{2\pi \sigma^{2}}}e^{-{\left(\frac{x-\mu }{\sqrt{2}\sigma }\right)}^{2}}-h\beta \frac{1}{\sqrt{2\pi \;\sigma^{2}}}e^{-{\left(\frac{x-\mu }{\sqrt{2}\sigma }\right)}^{2}}+\\ +h\beta {\left(x-\mu -\delta \right)}^{2}\frac{1}{\sqrt{2\pi \sigma^{2}}}e^{-{\left(\frac{x-\mu }{\sqrt{2}\sigma }\right)}^{2}} \end{array} \end{array}$$ (2)

where the first term is the main Gaussian, that models the overall vessel shape, the second term is the second Gaussian, that is subtracted to the first Gaussian and models the CLR, and the last is a Gaussian multiplied by a parabola, displaced in *x*, which controls the model asymmetry. Note that all the Gaussians have the same center and spread. The Gaussian which modulates the CLR has an independent amplitude. However, the third Gaussian, which is multiplied by the parabola, is also multiplied by the amplitude parameter of the CLR Gaussian. The effect of the values of the model parameters in the model shape is shown in Fig 8.

**Fig 9:**
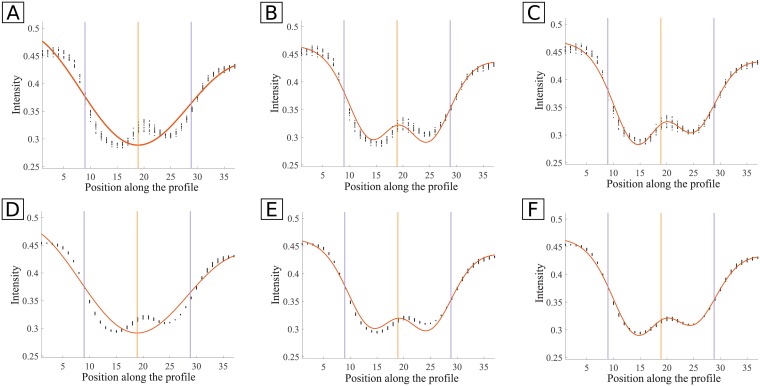
Examples of fitting of the Hermite, DoG-L7 and DoG-L8 models to smoothed and non-smoothed real vessel profiles. Profiles from 11 adjacent profiles are used. Black dots: profile data points; orange curve: fitted curve through Trust-Region-Reflective method; vertical yellow line: center of the profile; vertical purple lines: ground truth. **A**: original data, Hermite model (Eq 1); **B**: original data, DoG-L7 model (Eq 3); **C**: original data, DoG-L8 model (Eq 4); **D**: smoothed data, Hermite model; **E**: smoothed data, DoG-L7 model; **F**: smoothed data, DoG-L8 model.

One of the problems of this model is the fact that the second Gaussian (CLR) has the same spread as the main and the third Gaussians which in real vessel profiles is not necessarily true. In Fig 8B we can see this effect: when *σ* changes the spreads of all Gaussians change—since one single parameter is used -, thus forcing that wider vessels have wider CLR. Further, the fact that the two last Gaussian are multiplied by the same parameter (*β*) leads to restrictions in the fitting: the amplitudes of the CLR Gaussian and the third Gaussian are dependent. In Fig 8C the influence of changing *β* is shown, depicting the mentioned effect: changing the amplitude of the second Gaussian directly affects the amplitude of the third Gaussian. This means that we can not control the amplitude of the CLR independently. When comparing the curves with *β* = 1 and *β* = 0.2, for instance, we observe that the differences go beyond the change in amplitude of the CLR, being a consequence of the modification of the third Gaussian’s amplitude also. Additionally, by checking Fig 8D one can see that the modification of the *δ* parameter leads to more than the adjustment of the degree of asymmetry—it also affects greatly the amplitude of the final profile. Considering all these comments, we consider that, although addressing the existence of CLR and asymmetry in the vessel profile central region, the model parameters’ influence in the final profile are not that intuitive to capture and probably do not allow to cover all possible vessel profile shapes. An example of the fitting result to a vessel profile using the Hermite model is shown in Fig 9A and 9D. Although the model fits well simpler profiles, vessels with CLR are often poorly adjusted by this model.

#### 2.3.2 Modified DoG model with 7 parameters (DoG-L7)

**Fig 10:**
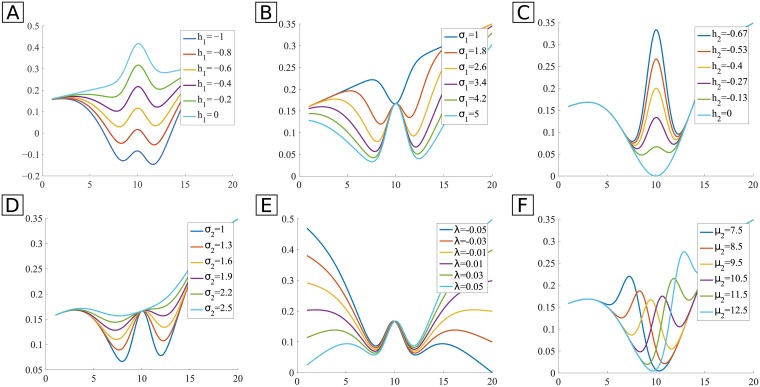
Examples of curves of the DoG-L7 (A-E) and DoG-L8 models (F), but in 1D. **A**: amplitude of the 1^st^ Gaussian (*h*_1_); **B**: spread of the 1^st^ Gaussian (*σ*_1_); **C**: amplitude of the 2^nd^ Gaussian (*h*_2_); **D**: spread of the 2^nd^ Gaussian (*σ*_2_); **E**: slope of the multiplying line (λ); **F**: center of the 2^nd^ Gaussian (*μ*_2_). A profile length of 20 pixels was set to the vessel. In each plot a parameter is varied (inside a established range) at a time, with the remaining parameters fixed (*t* = 0.5, *h*_1_ = −0.5, *μ* = 10, *σ*_1_ = 3, *h*_2_ = −0.33, *σ*_2_ = 1, λ = 0.02), in order to evaluate the influence of that parameter in the overall model shape.

A new model consisting in a modified Difference-of-Gaussians (DoG) is proposed. Although the common DoG model takes into account the CLR, it does not allow asymmetry between the vessel edges. This asymmetry is in fact present in some vessel profiles, which can lead to a poor fitting. This new model takes an adapted DoG (constrained in some parameters) and multiplies it by a line, in order to achieve the desired asymmetry in the vessel edges:

$$m_{1}\left(x,y\right)=\left(t+h_{1}e^{-{\left(\frac{x-\mu }{\sqrt{2}\sigma_{1}}\right)}^{2}}-h_{2}e^{-{\left(\frac{x-\mu }{\sqrt{2}\sigma_{2}}\right)}^{2}}\right)\left(\lambda \left(x-\mu \right)+t\right)$$ (3)

where *x* is the coordinate along the vessel cross-section, *t* is the maximum of the function, *h*_1_ is the height of the first (main) Gaussian, *μ* the location of the center, *σ*_1_ the spread of the first Gaussian, *h*_2_ the height of the second Gaussian, *σ*_2_ the spread of the second (CLR) Gaussian and λ is the slope of the multiplying line. As can be seen, the means of the two Gaussians are the same, centering the light reflex in the center of vessel. The effect of the values of the model parameters in the overall shape of the curve is shown in Fig 10. Comparing to what happened with the Hermite model, now there is a more clear relation between the parameters and their influence in the final profile. For instance, λ parameter (Fig 10E) just adjusts the inclination of the line that defines the asymmetry in the vessel profile edges. Note that his type of asymmetry is not the same as the considered in the Hermite model, where it was focused on the central part of the profile and not on their edges, as it is the case here. The amplitudes of the two Gaussians, *h*_1_ and *h*_2_, are two different parameters and thus independent (Fig 10A and 10C. Further, the spreads of the two Gaussians, *σ*_1_ and *σ*_2_ are also independent (Fig 10B and 10D). An example of the fitting result to a vessel profile using the DoG-L7 model is shown in Fig 9B and 9E.

#### 2.3.3 Modified DoG model with 8 parameters (DoG-L8)

Although the modified DoG model from Eq 3 behaves fairly well on the tested profiles, it does not allow to control in a good extent the asymmetry in the CLR. This means that in certain cases, such as the one shown in Fig 9, the central part of the vessel is not very well fitted by the model. To overcome this, another parameter is added to the model. Here, we choose to allow the CLR Gaussian to have a different mean from the main one:

$$m_{2}\left(x,y\right)=\left(t+h_{1}e^{-{\left(\frac{x-\mu_{1}}{\sqrt{2}\sigma_{1}}\right)}^{2}}-h_{2}e^{-{\left(\frac{x-\mu_{2}}{\sqrt{2}\sigma_{2}}\right)}^{2}}\right)\left(\lambda \left(x-\mu_{1}\right)+t\right)$$ (4)

where *μ*_1_ and *μ*_2_ are the center (mean) of the first and second Gaussians, respectively, and the other parameters have the same meaning as in Eq 3. This new parameter allows the displacement of the CLR Gaussian relatively to the main one, being able to model the desired asymmetry. The influence of this new parameter in the shape of the model is shown in Fig 10F. The profile can now have asymmetry in the central profile region—in a similar manner to what happened with Hermite model—and also on the profile limits—as happened with the DoG-L7 model. Fig 9C and 9F show the result of the fitting a profile using the DoG-L8 model. It is visible that the central region is now being very appropriately adjusted by the model curve.

Prior to fitting, the allowed range of parameters and the parameter initialization are defined based on the common appearance of the vessel profiles (S2 Appendix). In order to find the parameters of the best-fit model to the vessel profiles, a non-linear least squares problem is solved. The solution consists in the set of parameters that minimize the sum of the squared differences, defined as:

$$s\left(\beta \right)=\sum_{i=1}^{m}{\left[y_{i}-f\left(x_{i},\beta \right)\right]}^{2}$$ (5)

where (*x*_*i*_, *y*_*i*_) are empirical data pairs, *m* is the number of points, *f*(*x*, ***β***) is the model curve and ***β*** are parameters of the model curve.

The method used in this work to find the parameter values is the Trust-Region-Reflective [34], a region algorithm that is robust, reliable and has very strong convergence [35]. They have good performance, retrieving accurate results and being suitable for solving difficult nonlinear problems more efficiently than other algorithms.

### 2.4 Width estimation

Once the best-fit model to the vessel profile is found, the relationship between its parameters and the vessel width must be determined. In this work, this relation is found by using ensembles of bagged regression trees [8]. Ensemble methods, such as bagging, i.e., bootstrap aggregation, combine multiple weak trees, forming a more accurate and robust regressor than the individual trees [36]. We use, in fact, random forests [37, 38], where each tree in the ensemble can randomly select predictors for the decision splits, improving the accuracy of the predictions. Specifically, in this work we train the random forests having as input the *N* parameters of the model and as desired output the ground truth diameter. In this case, the regressor learns the mapping from a point in the *N*—dimensional parameter space, where *N* is the number of parameters of the model, to the vessel width. Then, the trained random forests can be used for outputting the estimated diameter value for a given test (i.e., never seen) profile, having as input the set of *N* parameters of the best-fit model to that profile.

## 3 Results

The conceived methodologies are evaluated in a publicly available dataset of annotated images. The experimental methodology for evaluating our approach is detailed in this section. The results of our method as well as from other state-of-the-art algorithms are presented and discussed.

### 3.1 REVIEW dataset

The Retinal Vessel Image set for Estimation of Widths (REVIEW) dataset [39] is the only public dataset with vessel width measurements, based on vessel edges marked by 3 observers on randomly selected segments using a special drawing tool. This dataset can be downloaded at http://ReviewDB.lincoln.ac.uk. REVIEW is the reference dataset for evaluation of width estimation algorithms in eye fundus images, and has been used by the majority of the state-of-the-art methods. It has 4 subsets, 16 images, 193 segments and 5066 profiles. These images have a variety of resolutions, pathologies and artifacts. The ground truth is the mean of the annotations of the 3 observers. The four subsets are: HRIS (The high resolution image set), VDIS (The vascular disease image set), CLRIS (The central light reflex image set) and KPIS (The kick point image set). The characteristics of these subsets are detailed in Table 1. Examples of images from REVIEW along with the ground truth markings are shown in S3 Appendix.

**Table 1:** REVIEW subsets characteristics. HRIS: The high resolution image set; VDIS: The vascular disease image set; CLRIS: The central light reflex image set; KPIS: The kick point image set (px: pixels; FOV: field of view; im: images; seg: segments; prof: profiles).

Dataset	size (pixels)	FOV	# im	# seg	# prof	notes
**HRIS**	2438×3584	60°	4	90	2368	different grades of DR; images usually sampled by a factor of 4 for algorithm evaluation
**VDIS**	1024×1360	50°	8	79	2249	normal and diseased retina (diabetic and atherosclerotic retinopathies
**CLRIS**	1440×2160	50°	2	21	285	early atherosclerotic changes with an exaggerated CLR
**KPIS**	288×119 and 170×192	60°	2	3	164	good quality images (retrieved from 2600×3330 pixel images); clean, large and non tortuous vessels

### 3.2 Evaluation metrics

For the current application, it is more relevant that the algorithms retrieve precise results, i.e., with a low standard deviation of the width errors, than accurate, i.e., low mean of the width errors [6], since any consistent bias can be compensated, whereas no compensation is possible for fluctuating bias. The standard deviation of the point-by-point differences between the measured and the ground truth diameters should thus be used to evaluate the performance of the algorithms [39]. This difference, at given vessel profile *i*, is given by χ_*i*_ = *ω*_*i*_ − *ψ*_*i*_, where *ω*_*i*_ is the estimated width and *ψ*_*i*_ is the correspondent ground truth. The standard deviation of the width differences is given by

$$\sigma_{error}=\sqrt{\frac{1}{n_{p}-1}\sum_{i=1}^{n_{p}}{\left(\chi_{i}-\mu_{error}\right)}^{2}}$$ (6)

where *μ*_*error*_ represents the mean of the width differences and is given by $\mu_{error}=\frac{1}{n_{p}}\sum_{i=1}^{n_{p}}\chi_{i}$, being *n*_*p*_ the number of vessel cross-sections, i.e., profiles, evaluated. The success rate (SR) is commonly used as a measure of stability [8]. It is usually defined as the ratio between the meaningful measurements returned by the algorithm and the total number of measurements. The mean and the standard deviation of the width measurements is also commonly reported.

In order to evaluate the performance of the algorithm, a correspondence has to be established between each ground truth center point (i.e., center point of the marked edge points) and a point in the detected centerline. Here, we associate each ground truth center point with the closest detected center point, as long as they are within less than 5 pixels from each other and that no other ground truth center point is closer to that detected point. This value is chosen since it is smaller than most of the diameters on the dataset and ensures some margin to account for possible mislocation of the detected center point. This leads to a unique match between ground truth and detected center points, ensuring that each center point is only used once for measurement. In the work of [11] a similar scheme is used, but a larger tolerance is given when it comes to the maximum distance between the two points (it has to be less than the true vessel diameter at that point). This strict criteria of unique matching between a ground truth and a detected point can lead to a decrease in the SR in cases where the ground truth points have a distance of less than 1 pixel from each other.

#### 3.2.1 Results per range of diameters

The analysis performance of the algorithm for different ranges of diameters is also performed. Ideally, the behaviour should be independent of the real vessel diameter, but some algorithms tend to retrieve worse results for a given range of diameters, generally for thinner vessels. One simple way to coarsely assess this is to compare the distributions of the measured and ground truth diameters. However, this does not retrieve information regarding the error for each range of diameters. Consequently, evaluation can be performed using Bland-Altman plots of the results, by plotting the differences between the measured and the ground truth widths (χ_*i*_) as a function of the mean of those differences.

#### 3.2.2 Goodness-of-fit

As the determination of the diameters is performed based solely on the model parameters,the goodness-of-fit of the model curves to the intensity cross-sectional profiles should be analysed, since the curves should represent the profile as accurately as possible, without compromising the performance of the regressor. Different metrics are herein used to evaluate this goodness-of-fit. The sum of squares due to error (SSE), also called sum of square of residuals, represents the deviation of the data points from the fitted curve. It is given by:

$$SSE=\sum_{i=1}^{n}{\left(y_{i}-\hat{y_{i}}\right)}^{2}$$ (7)

where *n* is the number of points in the profile, $\hat{y_{i}}$ the predicted, i.e., the model, value at point *i* and *y*_*i*_ the observation value. A smaller value, i.e., closer to zero, means that the model has a smaller random error, being more useful for prediction.

The R-square (R^2^) metric measures how well the fit explains the variation of the data, and is given by:

$$R^{2}=\frac{SSR}{SST}=1-\frac{SSE}{SST}$$ (8)

where SSR is the ratio of the sum of squares of the regression, $SSR=\sum_{i=1}^{n}\;{\left(\hat{y_{i}}-\bar{y}\right)}^{2}$, SST is the total sum of squares, $SST=\sum_{i=1}^{n}{\left(y_{i}-\bar{y}\right)}^{2}$, verifying *SST* = *SSR* + *SSE*, with $\bar{y}$ being the mean of the observations. It is also called the square of the correlation between the observation and the predicted values. *R*^2^ ranges from 0 to 1, with higher values indicating that the model accounts for a greater proportion of variance. Note that if the number of model coefficients increases, *R*^2^ increases without the fitting necessarily improving. To avoid this, the number of degrees of freedom should be accounted for (adjusted R-square). This adjusted R-square ($R_{adj}^{2}$) is given by:

$$R_{adj}^{2}=1-\frac{SSE(n-1)}{SST(v)}$$ (9)

where *v* is the number of residual degrees of freedom, *v* = *n* − *m*, with *n* being the number of data points and *m* the number of fitting coefficients. This metric can have any value smaller or equal to 1, being that values closer to 1 are indicative of a better fit.

Finally, the root mean squared error (RMSE), also called fit standard error, is defined as:

$$RMSE=\sqrt{MSE}=\sqrt{\frac{SSE}{v}}$$ (10)

RMSE values closer to 0 indicate a fit more useful for prediction.

### 3.3 Model fitting

**Fig 11:**
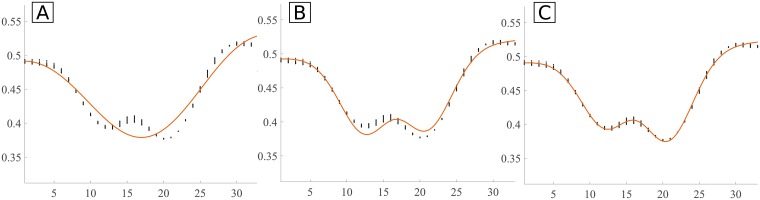
Example of fitting of the models to a vessel intensity profile. Black dots: profile; orange curve: best-fit model. **A**: Hermite model; *SSE* = 0.0586, *R*^2^ = 0.9329, $R_{adj}^{2}=0.9320$, *RMSE* = 0.0128; **B**: DoG-L7 model; *SSE* = 0.01524, *R*^2^ = 0.9825, $R_{adj}^{2}=0.9822$, *RMSE* = 0.0065; **C**: DoG-L8 model; *SSE* = 0.0030, *R*^2^ = 0.9965, $R_{adj}^{2}=0.9964$, *RMSE* = 0.0029.

The goodness-of-fit is evaluated for the three tested models: DoG×line model with 7 parameters (DoG-L7), DoG×line model with 8 parameters (DoG-L8) and the Hermite model with 6 parameters. The results of the goodness-of-fit (gof) metrics for each dataset of REVIEW are in Table 2. For each metric and dataset, it shows the mean of the metrics for all the profiles in that dataset. In Fig 11, examples of intensity profiles and their best-fit models are shown, along with the computed gof metrics.

**Table 2:** Goodness-of-fit metrics obtained for the DoG-L7, DoG-L8 and Hermite models. The results shown are the mean of the values from all the profiles of each dataset. The best results for each dataset are highlighted.

Model	Dataset	*SSE*	*R*^2^	$R_{adj}^{2}$	*RMSE*
**DoG-L7**	CLRIS	0.0072	0.9774	0.9769	0.0043
	HRIS	0.0075	0.9733	0.9721	0.0062
	KPIS	0.0055	0.9935	0.9932	0.0053
	VDIS	0.0072	0.9726	0.9718	0.0045
**DoG-L8**	CLRIS	**0.0053**	**0.9817**	**0.9813**	**0.0037**
	HRIS	**0.0071**	**0.9750**	**0.9736**	**0.0060**
	KPIS	**0.0050**	**0.9942**	**0.9940**	**0.0050**
	VDIS	**0.0064**	**0.9744**	**0,9735**	**0.0043**
**Hermite**	CLRIS	0.0412	0,9208	0.9197	0.0097
	HRIS	0.0090	0,9694	0.9683	0.0069
	KPIS	0.0110	0,9873	0.9870	0.0078
	VDIS	0.0114	0.9683	0.9675	0.0054

As it can be seen, the DoG-L8 model consistently returns better gof metrics than the other two models, followed by the DoG-L7. In fact, the 8 parameter model has always the lowest *SSE* and *RMSE* and the highest *R*^2^ and $R_{adj}^{2}$, which suggests it is the model with smaller random error and the one that better explains the variation of the data. This is specially noticeable for the CLRIS dataset, where the DoG-L8 model shows the largest improvement relatively to DoG-L7. This is expected since the new introduced parameter allows to model the asymmetry in the CLR, which is frequent in the CLRIS images. The Hermite model with 6 parameters is the worst fitting model, retrieving worse values for all the gof metrics, being more prominent in CLRIS.

### 3.4 Width measurement

The results of the method proposed in this work are evaluated in four different ways:

1. *k*-fold cross-validation, with *k* = 10, in each dataset (CLRIS, HRIS, KPIS and VDIS);
2. *k*-fold cross-validation, with *k* = 10, in the whole REVIEW dataset;
3. leave-one-segment-out validation, in each dataset;
4. leave-one-segment-out validation, in the whole REVIEW.

Tables 3 and 4 show the results of the different tested models, for each of the datasets of REVIEW, using each of the 4 referred evaluation schemes. The metrics presented in the table are the success rate of the algorithm, the mean and standard deviation of the measurements and the mean and standard deviation of the measurement errors. For comparison, results from the observers and the ground truth values are also shown. The goal here is not to achieve zero standard deviation of the errors, but instead to be close to the observers’ results.

**Table 3:** Results of the proposed method for retinal vessel width estimation using the DoG-L7, DoG-L8, and Hermite models in the CLRIS and HRIS datasets from REVIEW. Four evaluation schemes are presented: cross-validation in each dataset (Cv_d) and in the whole REVIEW (Cv_R) and leave-one-segment-out in each dataset (Lso_d) and in the whole REVIEW (Lso_R). O1, O2 and O3 are the observers, and G.T. is the ground truth, i.e., mean of the 3 observations. SR is the success rate, *μ*_*meas*_ and *σ*_*meas*_ are the mean and standard deviation of the width measurements, respectively, and *μ*_*error*_ and *σ*_*error*_ are the mean and standard deviation of the measurement errors. * SR values are negatively influenced by errors found in the observers’ annotations.

		CLRIS					HRIS				
Method		SR (%)	*μ*_*meas*_ (px)	*σ*_*meas*_ (px)	*μ*_*error*_ (px)	*σ*_*error*_ (px)	SR (%)	*μ*_*meas*_ (px)	*σ*_*meas*_ (px)	*μ*_*error*_ (px)	*σ*_*error*_ (px)
O1		100	13.19	4.01	-0.61	**0.566**	100	4.12	1.25	-0.23	**0.288**
O2		100	13.69	4.22	-0.11	**0.698**	100	4.35	1.35	0.002	**0.256**
O3		100	14.52	4.26	0.72	**0.566**	100	4.58	1.26	0.23	**0.285**
G.T.		100	13.80	4.12	-	-	100	4.35	1.26	-	-
DoG-L7	Cv_d	97.5*	13.78	3.90	0.01	**0.563**	98.4*	4.30	1.21	0.002	**0.217**
	Cv_R		13.61	3.92	-0.16	**0.685**		4.36	1.22	0.06	**0.302**
	Lso_d		13.56	3.64	-0.20	**1.236**		4.30	1.10	-0.004	**0.539**
	Lso_R		13.12	3.51	-0.64	**1.156**		4.53	1.32	0.23	**0.829**
DoG-L8	Cv_d	97.5*	13.78	3.93	0.01	**0.592**	98.4*	4.31	1.19	0.002	**0.248**
	Cv_R		13.43	3.90	-0.34	**0.796**		4.37	1.23	0.062	**0.320**
	Lso_d		13.63	3.68	-0.14	**1.297**		4.28	1.06	-0.03	**0.598**
	Lso_R		12.64	3.29	-1.12	**1.519**		4.49	1.35	0.19	**0.901**
Hermite	Cv_d	97.5*	13.78	3.62	0.02	**1.152**	98.4*	4.30	1.21	0.002	**0.221**
	Cv_R		13.52	3.23	-0.24	**1.391**		4.38	1.22	0.07	**0.287**
	Lso_d		13.70	3.24	-0.06	**2.306**		4.32	1.12	0.02	**0.488**
	Lso_R		12.92	2.59	-0.85	**2.752**		4.60	1.29	0.29	**0.718**

**Table 4:** Results of the proposed method for retinal vessel width estimation using the DoG-L7, DoG-L8, and Hermite models in the KPIS and VDIS datasets from REVIEW. Four evaluation schemes are presented: cross-validation in each dataset (Cv_d) and in the whole REVIEW (Cv_R) and leave-one-segment-out in each dataset (Lso_d) and in the whole REVIEW (Lso_R). O1, O2 and O3 are the observers, and G.T. is the ground truth, i.e., mean of the 3 observations. SR is the success rate, *μ*_*meas*_ and *σ*_*meas*_ are the mean and standard deviation of the width measurements, respectively, and *μ*_*error*_ and *σ*_*error*_ are the mean and standard deviation of the measurement errors. * SR values are negatively influenced by errors found in the observers’ annotations.

		KPIS					VDIS				
Method		SR (%)	*μ*_*meas*_ (px)	*σ*_*meas*_ (px)	*μ*_*error*_ (px)	*σ*_*error*_ (px)	SR (%)	*μ*_*meas*_ (px)	*σ*_*meas*_ (px)	*μ*_*error*_ (px)	*σ*_*error*_ (px)
O1		100	7.97	0.47	0.45	**0.233**	100	8.50	2.54	-0.35	**0.543**
O2		100	7.60	0.42	0.08	**0.213**	100	8.91	2.69	0.06	**0.621**
O3		100	7.00	0.52	-0.53	**0.234**	100	9.15	2.67	0.30	**0.669**
G.T.		100	7.52	0.42	-	-	100	8.85	2.57	-	-
DoG-L7	Cv_d	98.8	7.40	0.26	-0.001	**0.298**	99.7*	8.78	2.45	0.007	**0.690**
	Cv_R		7.34	0.29	-0.06	**0.299**		8.75	2.53	-0.02	**0.721**
	Lso_d		7.37	0.14	-0.03	**0.384**		8.69	2.25	-0.08	**1.048**
	Lso_R		7.16	0.27	-0.24	**0.365**		8.68	2.44	-0.09	**1.092**
DoG-L8	Cv_d	98.8	7.41	0.23	0.005	**0.304**	99.7*	8.79	2.39	0.016	**0.780**
	Cv_R		7.33	0.28	-0.08	**0.327**		8.78	2.49	0.008	**0.840**
	Lso_d		7.39	0.15	-0.001	**0.434**		8.62	2.10	-0.155	**1.234**
	Lso_R		7.12	0.26	-0.29	**0.408**		8.66	2.36	-0.11	**1.270**
Hermite	Cv_d	98.8	7.40	0.26	0.002	**0.283**	99.7*	8.77	2.41	0.001	**0.726**
	Cv_R		7.33	0.27	-0.07	**0.292**		8.72	2.51	-0.05	**0.785**
	Lso_d		7.42	0.17	0.02	**0.359**		8.66	2.24	-0.11	**1.099**
	Lso_R		7.18	0.22	-0.22	**0.384**		8.58	2.44	-0.19	**1.139**

#### 3.4.1 *k*-fold cross-validation results

In *k*-fold cross-validation, the original dataset is randomly partitioned in *k* subsets. At each time, *k* − 1 subsets are used for training and the remaining one for testing. This is repeated *k* times, so that each subset is used exactly one time for testing. This ensures that each profile enters exactly once for testing. In this work we use 10 folds, as in [8]. In [8] the authors perform cross-validation in each dataset. This means that for each of the 4 datasets in REVIEW, the cross-validation scheme is applied, independently of the other datasets. Here, we also perform cross-validation in the whole REVIEW. This allows to assess the robustness of the regression method, evaluating if it is able to return good results even when dealing with a large variety of images, both in terms of size, resolution, contrast, presence of pathologies, etc.

The results of the proposed method using the 3 tested models, evaluated through 10-fold cross-validation in each dataset and in the whole REVIEW, are shown in Tables 3 and 4. Results show to be close to the observers’ in terms of precision. The standard deviation of the errors, *σ*_*error*_, is consistently higher for CLRIS and VDIS datasets comparing to the HRIS and KPIS, which is coherent with the observers’ values. This is true across all the tested models, and for both cross-validation schemes. It is known that CLRIS is a difficult dataset due to the presence of accentuated CLR, and VDIS has a large variety of images, both normal and diseased, representing a greater challenge for diameter measurement.

Further, one notices that *σ*_*error*_ is generally slightly higher when performing cross-validation in the whole REVIEW than in each dataset. This effect is expected, since, despite the increase on the training set size that occurs when all datasets are considered, the variability of vessels properties also increases, as referred above. For instance, the range of diameters when performing the cross-validation in the whole REVIEW is larger than when each dataset is considered separately.

From the tested models, DoG-L7 shows better results. Although in the majority of the datasets the results of the three models are relatively close, for the CLRIS dataset the Hermite model behaves considerably worse, doubling *σ*_*error*_ of the other models. As referred in subsection 3.3, the fitting of the CLRIS profiles by this model is relatively poor, which negatively affects the results. Although the DoG-L8 model fits slightly better the CLR vessels (subsection 3.3), this improvement seems not to add much relevant information for regression. As can be seen, *σ*_*error*_ is similar for both DoG×line models in CLRIS, but for VDIS, for instance, it is higher for the 8 parameter model. This suggests that the addition of the parameter introduces extra information not relevant for the ensembles of bagged regression trees, which can even constitute noise that hinders the regression.

Regarding the success rates (SR), the major reason for the less than 100% SR of the proposed algorithm is the fact that no association is found between the dubious ground truth points (S4 Appendix) and the detected center points, due to the misplacement of the ground truth and the strict association criteria (subsection 3.2). In the case of KPIS, where no dubious ground truth marks were found, the profiles not measured correspond to junction regions. These junction points are removed in the algorithm’s preprocessing phase. It appears that some junctions were not avoided when marking the ground truth, probably due to the intersections with thin vessels that were not accounted for. However, as our segmentation detects even the thinnest vessels, these junctions are detected. Similar cases may occur in HRIS and VDIS datasets. Since our association criteria leads to a unique match between ground truth and detected center points, no association is performed at bifurcations and crossings. Despite this, considering the whole REVIEW, 99% of the ground truth vessel profiles are measured by the algorithm.

In S5 Appendix one can see that the measured diameters (DoG-L7 model) and the ground truth diameters follow a similar distribution. CLRIS is the dataset for which the distribution of the measurements is farther from the reference one. We can also see that the range of diameters present in the CLRIS and VDIS datasets are significantly larger that the ones from HRIS and KPIS, having KPIS the narrowest diameter range. Further, CLRIS is practically the only dataset that contains diameters over 15 pixels. HRIS, by its turn, contains very small diameters that are poorly represented in the other datasets. These facts help corroborating the obtained results, since the lower precision found in CLRIS and VDIS datasets when cross-validating in each dataset may be due to their broader diameter ranges. In fact, in [8] the authors suggest that when constructing datasets for ensembles of regression trees the distribution of the diameters in the training set should be approximately uniform and the range of widths in the training and testing sets should not be very wide.

**Fig 12:**
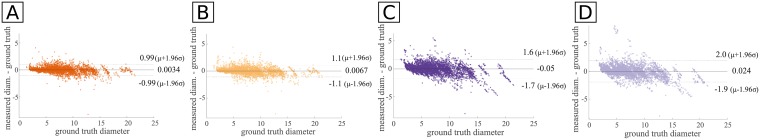
Bland-Altman plots of the ground truth and measured diameters, using the DoG-L7 model for fitting. Results for both 10-fold cross-validation and leave-one-segment-out validation, in each dataset (CLRIS, HRIS, KPIS and VDIS) and in the whole REVIEW, are shown (in the *xx* axis the ground truth diameters are plotted instead of the mean between the ground truth and measured diameters). **A**: cross-validation in each dataset; **B**: cross-validation in the whole REVIEW; **C**: leave-segment-out validation in each dataset; **D**: leave-segment-out validation in the whole REVIEW.

Fig 12A and 12B show the Bland-Altman plots of the ground truth and the measured diameters from REVIEW, using the DoG-L7 model and cross-validation in each dataset and in the whole REVIEW. In these plots, each point has coordinates (*x*_*p*_, *y*_*p*_ − *x*_*p*_), where *x*_*p*_ is the ground truth diameter and *y*_*p*_ the measured diameter. One can see that there is little dispersion of the points, indicating a small variance of the errors. As the points are close to the *y*_*p*_ − *x*_*p*_ = 0 line, a low measurement error is verified. Consequently, the measurements are both precise and accurate. The standard deviation of the errors for REVIEW is 0.51 pixels when the cross-validation is performed in each dataset, and 0.56 pixels when it is performed in the whole REVIEW, being the mean error close to zero. Further, the errors do not seem to depend on the range of diameters, since the points appear to be distributed in a similar manner regardless of the true diameter.

#### 3.4.2 Leave-one-segment-out validation results

The algorithm is also evaluated performing leave-one-segment-out validation. This consists in leaving out at each time one vessel segment for testing, and training in the remaining segments. The procedure is repeated *n* times, where *n* is the number of segments in the dataset. We evaluate our method in each dataset, as well as in the whole REVIEW. This way of evaluating the results ensures that similar neighboring profiles are not considered both in training and testing, which may not happen for the cross-validation scheme. Further, since 2D model fitting is performed, i.e., 11 neighboring profiles are considered, consecutive 2D profiles have 1D profiles in common.

The results of the proposed method using the 3 tested models and this evaluation scheme are shown in Tables 3 and 4. In general, the errors follow a tendency similar to the described for cross-validation, being verified higher *σ*_*error*_ for the CLRIS and VDIS datasets, for the three models and for the two validations (in-dataset and in the whole REVIEW). Similarly to what happened for cross-validation, DoG-L7 shows better or similar results to the ones from the other models, being the biggest improvement verified for CLRIS and when comparing with the Hermite model.

We see there is an increase of *σ*_*error*_ when using this validation instead of cross-validation, for all the datasets, which is expected. Removing an entire segment from the training set may significantly reduce or eliminate the presence of similar profiles to the tested ones. This effect is most prominent for the CLRIS dataset. As known, CLRIS contains segments with strong CLR, and has a very wide range of diameters. Further, it contains the majority of the vessels with diameters >20 pixels (S5 Appendix). Additionally, it only contains 20 segments. Since the diameter range is very wide, few segments are available for each diameter. Consequently, the removal of one segment from the training set can largely affect the ensembles of bagged regression trees since it is probably a representative segment. In S5 Appendix one sees that the measured diameter distribution does not have any profile with >20 pixels.

**Fig 13:**
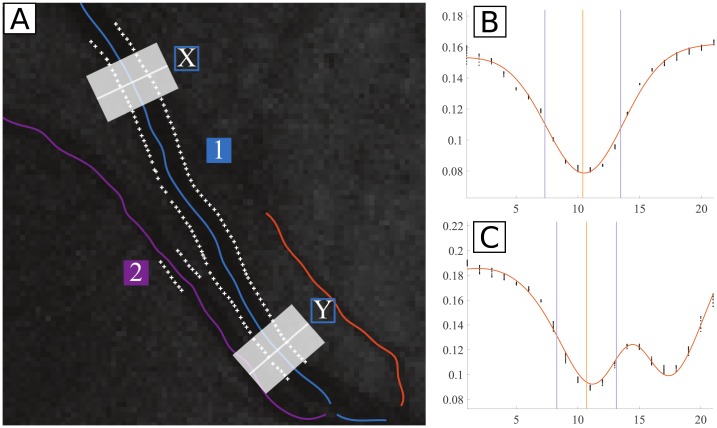
Example of poor width measurements due to a false central light reflex (CLR) detection. **A**: HRIS vessel segment (labeled as 1), that at a given point runs next to another segment (labeled as 2); **B**: smoothed profile extracted from region X; **C**: smoothed profile extracted from region Y, where the presence of another vessel close to the main vessel simulates the presence of CLR. Profiles as the one in [C] were wrongly measured by our algorithm when leave-one-segment-out validation in the whole REVIEW is performed, being retrieved a diameter that is approximately two times the real diameter. Black points in [B] and [C]: intensity profiles; orange curves: best-fit models, yellow vertical lines: centers of the profiles; purple vertical lines: ground truth locations; white marks in [A]: ground truth points.

Further, *σ*_*error*_ does not vary in the same manner for the different datasets when comparing both leave-one-segment-out validations. For HRIS, *σ*_*error*_ is higher (for all three models) when performing the validation in the whole REVIEW, whereas for the other datasets the differences are negligible. For the HRIS dataset, the diameter range is relatively narrow, and there are 90 segments in total (S5 Appendix). The fact that *σ*_*error*_ increases significantly when validating in the whole REVIEW is considered to be due to the introduction of noise by other datasets. Fig 13 shows a vessel profile from HRIS where the measured diameter was, for certain profiles, the double of the real one. This is caused by the similarity of the vessel profile with a profile of a vessel with CLR, caused by the inclusion of a near vessel in the profile. This identification of CLR only happened because of the presence of CLRIS in the training set.

Nevertheless, one should note that, even when a segment is left out of the training procedure, the obtained results are still very satisfactory, never surpassing approximately 1 pixel of *σ*_*error*_ (except for CLIRS, where *σ*_*error*_ is of 1.236 pixels). Finally, *μ*_*error*_ is considerably higher for the leave-one-segment-out validation in the whole REVIEW than for the other three validation schemes. Despite this, they are still close to the values of the observers.

An additional analysis is performed in order to assess the similarity between the segments in each dataset, aiming to corroborate the results of the leave-one-segment-out validation in each dataset. If the similarity between segments in a dataset is low, it is expected that the removal of a segment from the training set significantly affects the results. Obviously, this depends also on the number of segments in the dataset. To assess the segment similarity, for each dataset, the correlation between each pair of segments is computed. For that, the profile for a given segment is taken as the mean of the profiles from that segment. For each segment pair, the two profiles are aligned by their maximum value. Results are normalized by dividing by the maximum of the autocorrelations of the two profiles. Results are shown in Table 5. KPIS is the dataset that shows highest correlation between its segments (higher *μ*_*corr*_ and smaller *σ*_*corr*_), which is coherent with the evolution of *σ*_*error*_ when a segment is left out of the training, which is not very significant. CLRIS dataset shows one of the lowest correlations, which corroborates the big effect of the removal of a segment from the training set. Although VDIS has lower correlation values, the largest number of segments (79 vs 20) justifies the smaller increase in *σ*_*error*_ when comparing to CLRIS.

**Table 5:** Correlation between the segments of each dataset. *μ*_*corr*_ and *σ*_*corrr*_ are the mean and standard deviation of the correlations of all pairs of segments, *max*_*corr*_ is the maximum correlation and #*comb* is the number of combinations of 2 segments found in the dataset.

Dataset	*μ*_*corr*_	*σ*_*corr*_	*max*_*corr*_	#*comb*
**CLRIS**	0.510	0.252	0.982	190
**HRIS**	0.612	0.217	0.996	3160
**KPIS**	0.804	0.103	0.960	15
**VDIS**	0.454	0.258	0.997	6670

Fig 12C and 12D show the Bland-Altman plots of the ground truth and the measured diameters from REVIEW, using the DoG-L7 model and leave-one-segment-out in each dataset and in the whole REVIEW. Although there is little dispersion of the points, indicating a small variance of the errors, the dispersion is larger than that of cross-validation results. The standard deviation of the errors for REVIEW is 0.84 pixels when the cross-validation is performed in each dataset, and 0.99 pixels when it is performed in the whole REVIEW, being the mean error close to zero. Similarly to cross-validation, the errors do not seem to depend on the range of diameters, although a slight tendency to underestimate the widths for larger vessels can be detected.

### 3.5 Comparison with the state-of-the-art

Together with our DoG-L7 and DoG-L8-based methods, Table 6 depicts the performance of some of the methods in the literature on the REVIEW dataset in terms of standard deviation of the error. Additionally, in S6 Appendix the mean of the errors and the success rate of these methods are presented. Note that the evaluation of the methods that are not herein compared is not available in the literature. From the analysis of the state-of-the-art results, the majority of the methods tend to underestimate the widths (mean of the measurement errors ≤ 0). The best results, considering both accuracy and precision, usually occur for the HRIS and KPIS datasets. Some of the methods, as the earlier methods and that of [40], see their performance reduced in CLRIS dataset, namely in terms of precision and success rate. Generally, the most recent methods (from 2009 onwards) have more promising results, showing higher precision and accuracy. The most robust state-of-the-art algorithms are the ones from [9, 11] and [8], being those that return consistently low standard deviation of the errors for all 4 datasets.

**Table 6:** Standard deviation of the width errors for each of the four REVIEW datasets. The width errors are the point-by-point differences between the ground truth and the width measurements (pixels). Cv_d, Cv_R, Lso_d and Lso_R stand for cross-validation in the dataset and in the whole REVIEW, and leave-one-segment-out validation in the dataset and in the whole REVIEW, respectively. The score is the mean of the values of all datasets. The 3 best scores at highlighted.

Method	HRIS	VDIS	CLRIS	KPIS	Score
O1	0.288	0.543	0.567	0.233	0.408
O2	0.256	0.621	0.698	0.213	0.447
O3	0.285	0.669	0.566	0.234	0.439
Gregson [41]	1.479	1.494	2.841	0.602	1.604
HHFW [42]	0.926	0.879	-	0.389	-
Zhou (1D-G) [14]	0.896	2.110	4.137	0.399	1.886
Lowell (2D-G) [6]	0.703	1.328	6.019	0.337	2.097
Al-Diri (ESP) [9]	0.420	0.766	1.469	0.328	0.746
Yin, Y. [12]	-	-	-	-	-
Xu (Graphs) [10]	0.567	1.43	1.78	0.67	1.112
Trucco [43]	0.760	1.381	1.229	0.319	0.922
Kumar (ULDM) [7]	0.79	1.18	1.79	0.60	1.090
Lupascu [8]	0.438	1.073	1.154	0.318	0.746
Bankhead [11]	0.32	0.95	0.95	0.29	**0.628**
Yin, X. [44]	1.11	1.55	1.49	1.32	1.368
Vazquez- G [40]	0.85	1.11	2.17	0.76	1.223
Vazquez- L [40]	0.88	1.08	4.26	0.74	1.740
Vazquez- J [40]	0.80	1.19	2.30	0.73	1.255
Vazquez- I [40]	0.96	1.12	2.51	0.75	1.335
Proposed, DoG-L7 (Cv_d)	0.217	0.690	0.563	0.298	**0.442**
Proposed, DoG-L7 (Cv_R)	0.302	0.721	0.685	0.299	**0.502**
Proposed, DoG-L7 (Lso_d)	0.539	1.048	1.236	0.384	0.802
Proposed, DoG-L7 (Lso_R)	0.829	1.092	1.156	0.365	0.860
Proposed, DoG-L8 (Cv_d)	0.248	0.780	0.592	0.304	**0.481**
Proposed, DoG-L8 (Cv_R)	0.320	0.840	0.796	0.327	**0.571**
Proposed, DoG-L8 (Lso_d)	0.598	1.234	1.297	0.434	0.891
Proposed, DoG-L8 (Lso_R)	0.901	1.270	1.519	0.408	1.204

Our method shows, in general, the best performance, having the lowest *σ*_*error*_ when cross-validation is performed, both in each dataset and in the whole REVIEW. Even with other validation schemes, our results are among the best found in the literature. Considering a score defined as the mean of the standard deviation of the errors for all datasets, our DoG-L7-based method presents scores of 0.442 pixels and 0.502 pixels when using cross-validation in each dataset and in the whole REVIEW, respectively. When using the DoG-L8 model, the scores are 0.481 pixels and 0.571 pixels, respectively. The work of [11] has the third best score (0.628 pixels). When leave-one-segment-out is performed, our method is still among the three best scored-works (see Table 6).

Regarding the method of [8], which is conceptually closest to our proposal, as it performs Hermite model fitting and regression for width estimation, its performance is evaluated through cross-validation in each dataset. Using the same evaluation scheme, our method achieves almost half of the *σ*_*error*_ for the majority of the datasets. The improvement of the results can be attributed to the use of a model that fits best the vessel profiles (DoG-L7 and DoG-L8), specially those with CLR, and to the preparation of the profiles before model fitting using several preprocessing steps that improved the subsequent steps. This is true since the results of our method (see Tables 3 and 4) using the Hermite model with 6 parameters of [8] are still superior to those of that work for the majority of the datasets.

## 4 Conclusions

The method herein presented for vessel width measurement in retinal images combines model fitting with several preprocessing steps, and estimates the widths based on the best-fit-model parameters using ensembles of bagged regression trees with random feature selection. It uses a novel parametric model based on a Difference-of-Gaussians (DoG) model, modified through a multiplying line with varying inclination which is able to describe profile asymmetry.

Our method often shows better results than the top-performing state-of-the art algorithms. It has consistently the higher precision (lowest standard deviation of the errors) when cross-validation is performed. When a segment is left out, our results are still among the best found in the literature. Our method practically halves the standard deviation of the errors reported by [8]. The novel DoG-L7 and DoG-L8 models fit best the vessel profiles, specially the most challenging ones, such as the ones with central light reflex (CLR). Results are further improved due to the use of several preprocessing steps before model fitting.

Despite all this, there is still room for improvement and adaptations. The method was designed independently of any framework. However, depending on the future application, it could be of interest to adapt the width measurement algorithm to be autonomous from the vessel centerlines.

Further, considering the nature of the method used for width estimation, which relies on supervised learning, the definition of the training dataset is a key factor. However, the REVIEW dataset, as well as its sub-datasets, does not contain an uniform distribution of the diameters. This is not desirable and can influence the ensembles’ performance, being that a more balanced dataset would be of much more interest.

Additional efforts in optimizing certain steps of the algorithm would probably improve the results. For instance, the determination of the profile lengths prior to model fitting could be further refined, since a better initial width estimation could improve the model fitting results. Although the parameter choice is not very determinant to the ensembles of trees’ performance, the influence of the parameters, could be further assessed. Further, other regression methods could be tested for results comparison, such as Support Vector Machines or Neural Networks for regression.

Our retinal vessel width measurement method has a performance that is close or outperforms the top-performing state-of-the-art methods. The method shows to retrieve precise results, close to that of the observers, as was the goal. This shows the robustness of our method and its great potential to be used directly for measurement of retinal vessel widths and/or to be integrated in a framework for retinal vascular assessment.

## Supporting information

S1 Appendix Vessels with and without central light reflex. Examples of vessels with and without central light reflex and the respective profiles.
(PDF)

S2 Appendix Parameter ranges and initialization. Values for parameter initialization and range definition for performing model fitting.
(PDF)

S3 Appendix Examples of REVIEW images and markings. Eye fundus images selected from the REVIEW dataset, along with the respective ground truth markings.
(PDF)

S4 Appendix Dubious markings on REVIEW images. Examples of dubious markings from the observers on images from the REVIEW dataset.
(PDF)

S5 Appendix Diameter distributions. Distribution of the ground truth and measured diameters, using the DoG-L7 model for fitting and 10-fold cross-validation and leave-one-segment-out validation, both in each dataset and in the whole REVIEW.
(PDF)

S6 Appendix Methods’ results in REVIEW. Mean of the width errors and success rate of the proposed method and of state-of-the-art methods on the REVIEW dataset.
(PDF)

S1 Video Developed Graphical User Interface. Demo of the developed graphical user interface for the proposed retinal vessel width estimation method.
(MP4)
