# Supplementary material for: Parametric model fitting-based approach for retinal blood vessel caliber estimation in eye fundus images
Source: PLoS One. 2018 Apr 18;13(4):e0194702. doi: 10.1371/journal.pone.0194702 (PMC5905988; doi:10.1371/journal.pone.0194702)
Supplement: S1 Appendix — Examples of vessels with and without central light reflex and the respective profiles. (PDF) [file pone.0194702.s001.pdf]

Vessels with and without central light reflex

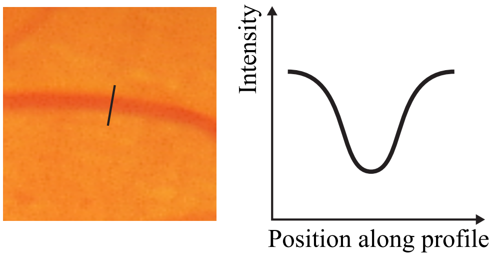

(a) Vessel without CLR and a profile taken from the vessel.

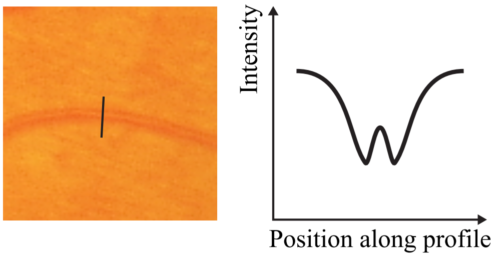

(b) Vessel width CLR and a profile taken from the vessel.

Figure 1: Vessel profiles with and without central light reflex (CLR) in a fundus image. Vessel profiles commonly present a Gaussian-like shape, with the minimum intensity occurring approximately in the vessel center region. Large vessels can present CLR, that appears as an increase of intensity in the center of the vessel, i.e., an elevation in the center of the vessel profile. This phenomena is caused by specular reflection and happens more frequently in digital fundus images than in fluorescein images.
