# Supplementary material for: Parametric model fitting-based approach for retinal blood vessel caliber estimation in eye fundus images
Source: PLoS One. 2018 Apr 18;13(4):e0194702. doi: 10.1371/journal.pone.0194702 (PMC5905988; doi:10.1371/journal.pone.0194702)
Supplement: S2 Appendix — Values for parameter initialization and range definition for performing model fitting. (PDF) [file pone.0194702.s002.pdf]

Parameter ranges and initialization

For the Hermite model, the same parameter ranges and initialization of the parameters from Lupascu *et al.* (2013) are used:

- $t \in [0, 1]$, t_{init}=max($mean\_profs$);
- $h \in [-0.588, 0]$, h_{init}=$-$(max($mean\_profs$)$-$min($mean\_profs$));
- $\beta \in [-1, 1]$, β_{init}=0;
- $\mu \in [0, prof\_length]$, μ_{init}=$center\_prof$;
- $\delta \in [-2, 2]$, δ_{init}=0.2;
- $\sigma \in [1, 15]$, σ_{init}=$prof\_length$/std($mean\_profs$),

where $mean\_profs$ is the mean of the adjacent profiles used in the 2D fitting, $center\_prof$ is the estimated center of the profile and $prof\_length$ is the estimated length of the profile.

Regarding the proposed models, the optimum range of values for each parameter is defined based on the known relation between some of the parameters and the common vessel profiles. To confirm that the ranges were broad enough, the observation of a large number of vessel intensity profiles was performed. The chosen parameter ranges and initialization for the modified DoG model with 7 parameters are the following:

- $t \in [0, 1]$, t_{init}=max($mean\_profs$);
- $h_1 \in [-1, 0]$, $h_{1_{init}}$=$-$(max($mean\_profs$)$-$min($mean\_profs$));
- $\mu \in [0, prof\_length]$, μ_{init}=$center\_prof$;
- $h_2 \in [-1/1.5, 0]$, $h_{2_{init}}$=$-1/3$;
- $\sigma_1 \in [1, prof\_length/4]$, $\sigma_{1_{init}}$=$(1+prof\_length/4)/2$;
- $\sigma_2 \in [1, prof\_length/8]$, $\sigma_{2_{init}}$=$\sigma_{1_{init}}/3$;
- $\lambda \in [-1/prof\_length, 1/prof\_length]$, λ_{init}=0,

where $mean\_profs$ is the mean of the adjacent profiles used in the 2D fitting, $center\_prof$ is the estimated center of the profile and $prof\_length$ is the estimated length of the profile. For the 8 parameter model, the parameter ranges are the same as for the other DoG model, and the additional parameter, μ_2, has the same range and initialization as μ.
