# Supplementary material for: Parametric model fitting-based approach for retinal blood vessel caliber estimation in eye fundus images
Source: PLoS One. 2018 Apr 18;13(4):e0194702. doi: 10.1371/journal.pone.0194702 (PMC5905988; doi:10.1371/journal.pone.0194702)
Supplement: S3 Appendix — Eye fundus images selected from the REVIEW dataset, along with the respective ground truth markings. (PDF) [file pone.0194702.s003.pdf]

Examples of REVIEW images and markings

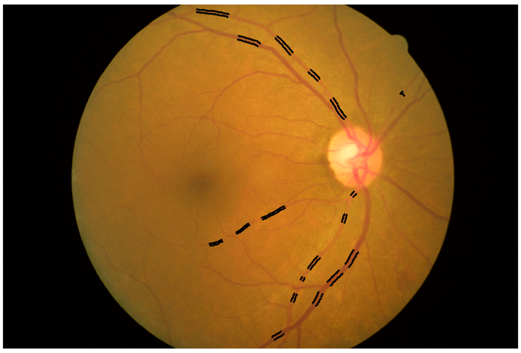

(a) CLRIS dataset (image CLRIS002).

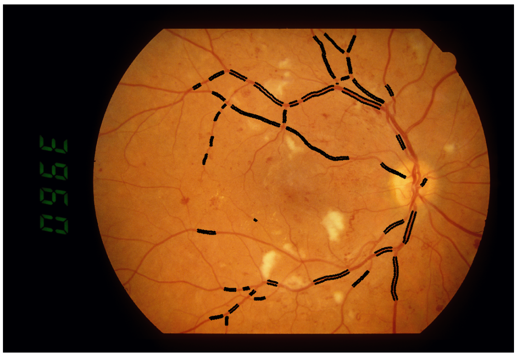

(b) HRIS dataset (image HRIS001).

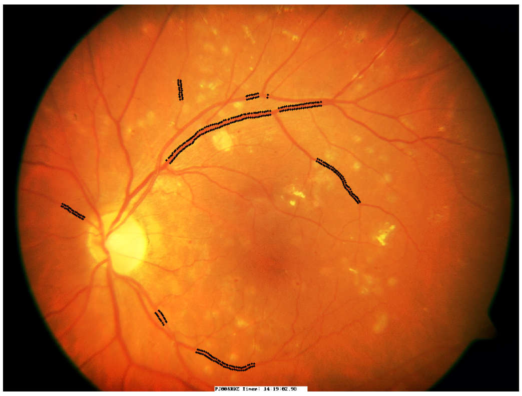

(c) VDIS dataset (image VDIS005).

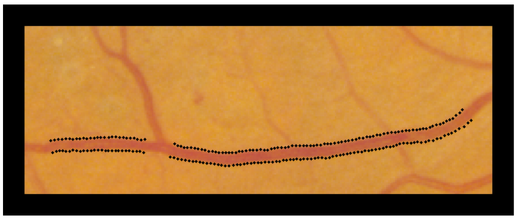

(d) KPIS dataset (image KPIS001).

Figure 1: Eye fundus images selected from the REVIEW dataset. Black marks represent the ground truth edge points defining the vessel width (mean of the annotations from the 3 observers).
