# Supplementary material for: Parametric model fitting-based approach for retinal blood vessel caliber estimation in eye fundus images
Source: PLoS One. 2018 Apr 18;13(4):e0194702. doi: 10.1371/journal.pone.0194702 (PMC5905988; doi:10.1371/journal.pone.0194702)
Supplement: S4 Appendix — Examples of dubious markings from the observers on images from the REVIEW dataset. (PDF) [file pone.0194702.s004.pdf]

Dubious markings on REVIEW images

There are cases of ground truth marks that look dubious since they are not close to the edges of the vessels. Fig. 1 shows examples of the cases we found where the ground truth does not seem correct. These images show the marks, at different colors, of the three observers. As shown in Fig. 1b, in some cases the marks of one of the observers are displaced relatively to the other two, leading to a wrong ground truth since the mean of the three does not return points on the edges. Besides, in one of the cases the marks of one observer are not placed along the vessel at some points (Fig. 1a). However, to our knowledge, there are no references regarding this problem in other works.

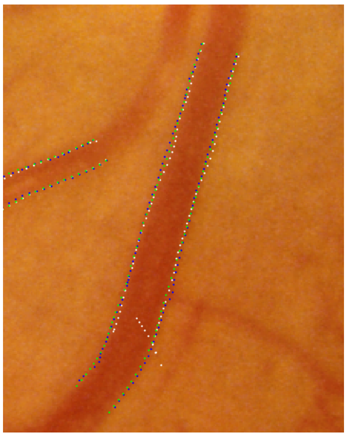

(a) HRIS001 (REVIEW).

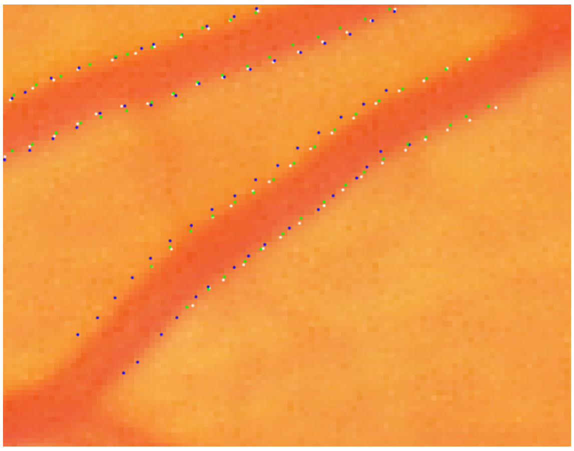

(b) VDIS006 (REVIEW).

Figure 1: Examples of dubious markings on images from the REVIEW dataset. The images shown correspond to the regions of the original images where the erroneous markings were detected. The marks represent the three observers' annotations, distinguishable by color.
