# Supplementary material for: Parametric model fitting-based approach for retinal blood vessel caliber estimation in eye fundus images
Source: PLoS One. 2018 Apr 18;13(4):e0194702. doi: 10.1371/journal.pone.0194702 (PMC5905988; doi:10.1371/journal.pone.0194702)
Supplement: S5 Appendix — Distribution of the ground truth and measured diameters, using the DoG-L7 model for fitting and 10-fold cross-validation and leave-one-segment-out validation, both in each dataset and in the whole REVIEW. (PDF) [file pone.0194702.s005.pdf]

Diameter distributions

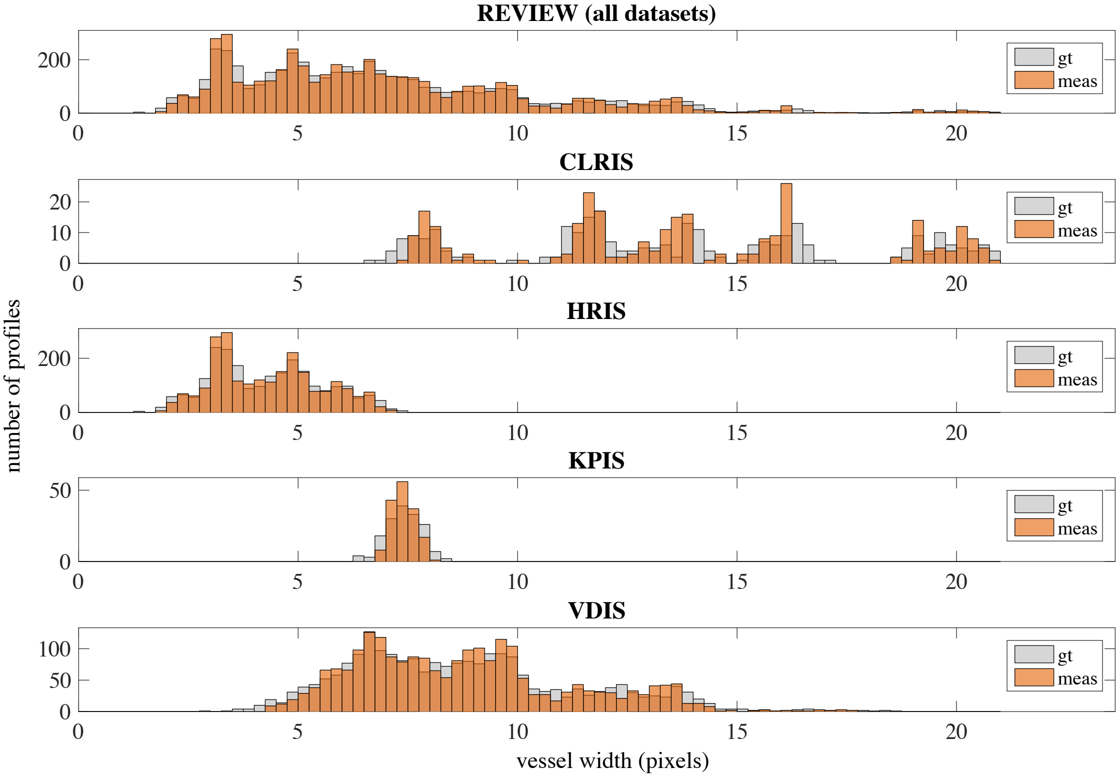

(a) Diameter distribution using cross-validation in each dataset (CLRIS, HRIS, KPIS and VDIS).

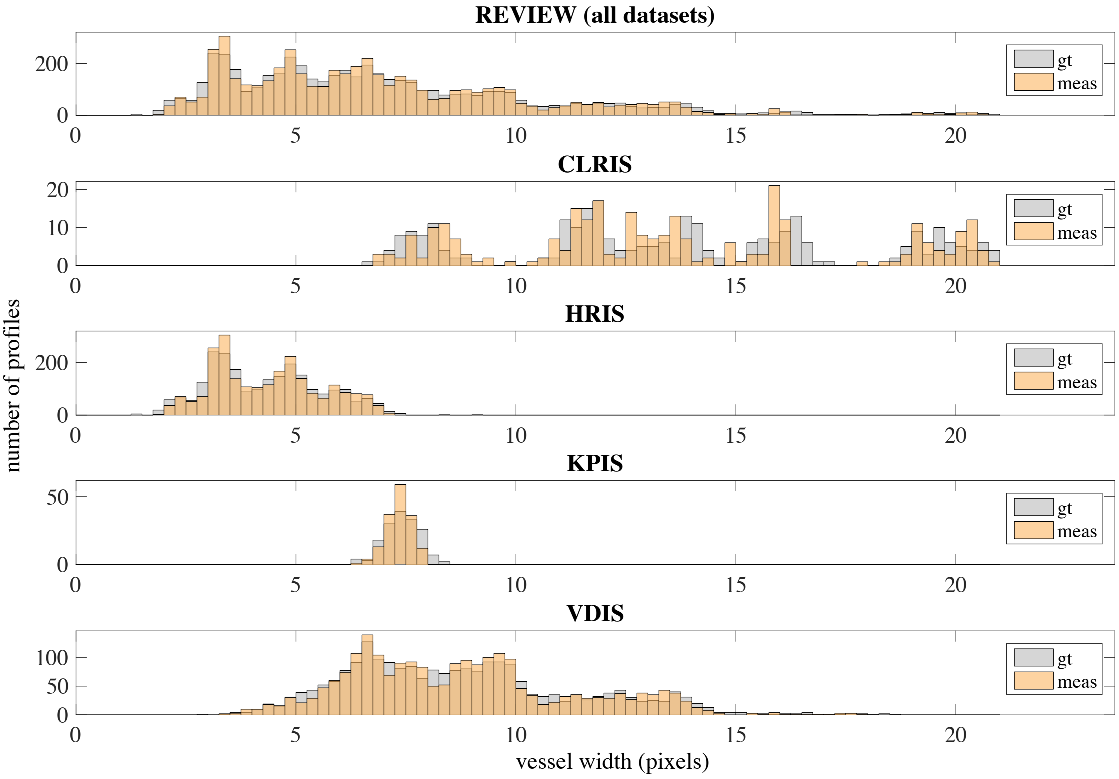

(b) Diameter distribution using cross-validation in the whole REVIEW.

Figure 1: Distribution of the ground truth and measured diameters, using the DoG-L7 model for fitting and 10-fold cross-validation, both inside each dataset and in the whole REVIEW.

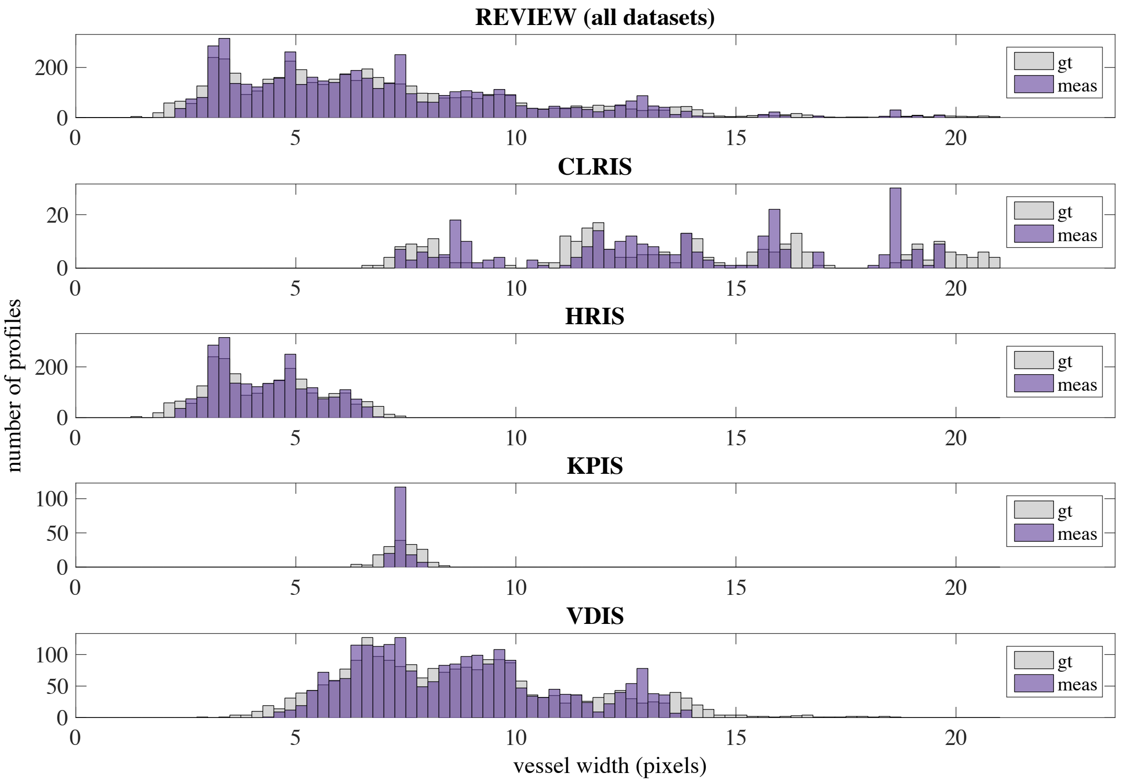

(a) Diameter distribution using leave-one-segment-out validation inside each dataset.

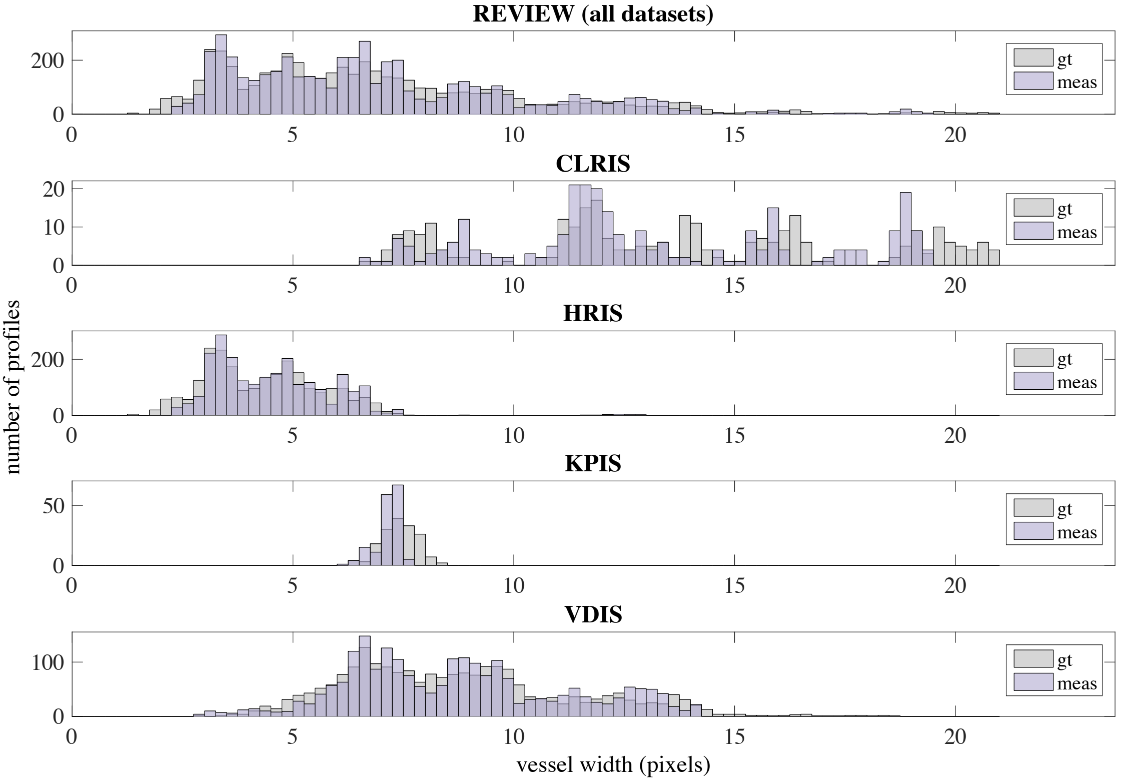

(b) Diameter distribution using leave-one-segment-out validation inside the whole REVIEW.

Figure 2: Distribution of the ground truth and measured diameters, using the DoG-L7 model for fitting and leave-one-segment-out validation, both inside each dataset and in the whole REVIEW.
