# Supplementary material for: Parametric model fitting-based approach for retinal blood vessel caliber estimation in eye fundus images
Source: PLoS One. 2018 Apr 18;13(4):e0194702. doi: 10.1371/journal.pone.0194702 (PMC5905988; doi:10.1371/journal.pone.0194702)
Supplement: S6 Appendix — Mean of the width errors and success rate of the proposed method and of state-of-the-art methods on the REVIEW dataset. (PDF) [file pone.0194702.s006.pdf]

Methods' results in REVIEW

Table 1: Mean of the width errors, i.e., point-by-point differences between the ground truth and the obtained width measurements (pixels). Cv_d, Cv_R, Lso_d and Lso_R stand for cross-validation in the dataset, cross-validation in the whole REVIEW, leave-one-segment-out validation in the dataset and leave-one-segment-out in the whole REVIEW, respectively.

Method	HRIS	VDIS	CLRIS	KPIS
O1	−0.23	−0.35	−0.61	0.45
O2	0.002	0.06	−0.11	0.08
O3	0.23	0.30	0.72	−0.53
Gregson	3.29	1.22	−1.0	−0.23
HHFW	0.62	−0.91	-	−1.05
Zhou (1D-G)	−0.54	−3.07	−7.5	−2.57
Lowell (2D-G)	−0.17	−2.26	−6.8	−1.65
Al-Diri (ESP)	0.28	−0.05	−1.90	−0.96
Yin, Y. (2013)	-	-	-	-
Xu (Graphs)	0.21	−0.53	0.08	−1.14
Trucco	−0.42	−0.79	−0.16	−1.32
Kumar (ULDM)	0.21	−0.64	−0.55	−0.50
Lupascu	0.004	0.015	0.006	0.015
Bankhead	-	-	-	-
Yin, X. (2014)	-	-	-	-
Vazquez- G	−0.13	−0.95	0.83	−1.28
Vazquez- L	−0.22	−1.29	1.20	−1.33
Vazquez- J	0.08	−0.84	1.52	−1.05
Vazquez- I	−0.14	−1.17	1.27	−1.26
Proposed, DoG-L7 (Cv_d)	0.002	0.007	0.01	−0.001
Proposed, DoG-L7 (Cv_R)	0.06	−0.02	−0.16	−0.06
Proposed, DoG-L7 (Lso_d)	−0.004	−0.08	−0.20	−0.03
Proposed, DoG-L7 (Lso_R)	0.23	−0.09	−0.64	−0.24
Proposed, DoG-L8 (Cv_d)	0.002	0.016	0.01	0.005
Proposed, DoG-L8 (Cv_R)	0.062	0.008	−0.34	−0.08
Proposed, DoG-L8 (Lso_d)	−0.03	−0.155	−0.14	−0.001
Proposed, DoG-L8 (Lso_R)	0.19	−0.11	−1.12	−0.29

Table 2: Success rate (%).

Method	HRIS	VDIS	CLRIS	KPIS
O1	100	100	100	100
O2	100	100	100	100
O3	100	100	100	100
Gregson	100	100	100	100
HHFW	88.3	78.4	0	96.3
Zhou (1D-G)	99.6	99.9	98.6	100
Lowell (2D-G)	98.9	77.2	26.7	100
Al-Diri (ESP)	99.7	99.6	93.0	100
Yin, Y.	100	100	100	100
Xu (Graphs)	100	96.0	94.1	99.4
Trucco	95.7	92.1	90.2	93.9
Kumar (ULDM)	99.6	96.3	98.2	100
Lupascu	100	100	100	100
Bankhead	99.5	99.0	100	100
Yin, X.	100	100	100	100
Vazquez- G	78.89	78.70	91.58	100
Vazquez- L	81.71	69.68	74.39	100
Vazquez- J	73.86	57.80	80.35	100
Vazquez- I	83.36	74.34	75.79	100
Proposed method	98.4	99.7	97.5	98.8
